# Systematic Review on the Effects of Prompt Antibiotic Treatment on Survival in Septic Shock and Sepsis Patients in Different Hospital Settings

**DOI:** 10.7759/cureus.32405

**Published:** 2022-12-11

**Authors:** Dania A Al-Kader, Sana Anwar, Helai Hussaini, Emilia E Jones Amaowei, Sayed Farhad Rasuli, Nabeel Hussain, Saleh Kaddo, Asadullah Memon

**Affiliations:** 1 Radiology, Shar Hospital, Sulaymaniyah, IRQ; 2 Internal Medicine, Liaquatian Research Council, Hyderabad, PAK; 3 Clinical Affairs, Dexcom Inc, San Diego, USA; 4 Basic Sciences, Liaquatian Research Council, Hyderabad, PAK; 5 Family Medicine, Liaquatian Research Council, Hyderabad, PAK; 6 Emergency Department, Sindh Government Hospital Kohsar, Hyderabad, PAK

**Keywords:** antibiotic administration in management of severe sepsis, sepsis, septic shock management, mortality rate in sepsis, time to antibiotic administration

## Abstract

This study aims to determine the impact of prompt administration of antibiotics in evaluating the prognosis of patients with septic shock or sepsis. On January 1, 2022, we searched the Cochrane Library, EMBASE, and MEDLINE databases for English-language articles regarding when antibiotics should be administered to patients with septic shock or sepsis. These articles were required to be published between 2010 and 2021. The primary objective was sudden or expected death from any cause at a specified time. In the study, 154,330 patients from 35 sepsis trials were included. In 19 trials, the effectiveness of antibiotics administered to 20,062 patients was evaluated. Of those, 16,652 received the correct medications. In 24 studies, the length of time it took to administer antibiotics was associated with an increased mortality rate. In fourteen studies, the time limits associated with patient outcomes ranged from 1 to 125 minutes to three to six hours. In eight studies, there were hourly delays, and in two, the time it took to receive an antibiotic played a role. Separately analyzed, the outcomes for septic shock (12,756 patients in 11 trials) and sepsis (24,282 patients in six studies) were identical. Two-thirds of sepsis studies discovered a correlation between early antibiotic treatment and the patient's prognosis. However, antimicrobial timing metrics varied significantly between studies, and there were no clear time limits.

## Introduction and background

Each year, approximately 11 million people die from sepsis, out of an estimated 49 million cases worldwide. Those with sepsis have an approximate 10% mortality rate, whereas those in septic shock have a mortality rate of over 40% [1]. Important to sepsis treatment protocols is the use of effective antibiotic therapy. However, there is considerable debate regarding how quickly antibiotics should be administered to sepsis patients. Researchers have demonstrated that prompt medical treatment of sepsis may improve patient survival [2]. Although some studies have found an association between early antibiotic treatment and a better prognosis, others have not [3-5]. According to research published in 2006 by Kumar et al., every hour that septic shock goes untreated increases mortality by 7.6% [6]. Some follow-up studies were unable to confirm these results, while others demonstrated a correlation between antibiotic treatment delay and an increase in mortality. Nonetheless, a number of studies [7] have found no correlation between antibiotic delivery delays and an increase in mortality. Patients with sepsis or septic shock should receive intravenous antimicrobials within the first hour of diagnosis, as recommended by the Surviving Sepsis Campaign (SSC) [8]. One such proposal from the SSC is a "sepsis bundle" mandating the use of broad-spectrum antibiotics within three hours of triage in the emergency department [9]. Nevertheless, the authors of the SSC procedures acknowledge that it is possible that these objectives will not always be met and that previous research has demonstrated that compliance with standards governing antimicrobial therapy is not always achieved. Antibiotic use as a quality-of-care indicator for patients with severe sepsis and septic shock has piqued interest despite significant limitations. As a result, we decided to conduct a literature review on the influence of antimicrobial medication timeliness on the outcome of sepsis-diagnosed patients in order to determine the relationship between antimicrobial medication timing and improved outcomes.

## Review

Methodology

Search Strategy

On January 1, 2022, we searched EMBASE, the Cochrane Library, and MEDLINE for relevant English-language articles published between 2010 and 2021. In addition, we searched the US National Institutes of Health Ongoing Trials Register ClinicalTrials.gov and Open-SIGLE databases for unpublished research and conference proceedings between 2008 and 2020. To find additional relevant research, we examined the sources cited in the publications. The search criteria included terms from the following Medical Subject Headings: (severe sepsis OR Sepsis OR septic shock) AND (antibiotics OR antibacterial agents OR antimicrobial agents) AND (time-to-antibiotic OR treatment timing).

Data Extraction

The writer conducted a full-text analysis on the publications that passed the first abstract screening. We included studies of patients over the age of 18 who were diagnosed with septic shock, severe sepsis, or sepsis according to the consensus conference definitions [10,11]. We hypothesized that participants with "severe sepsis" and participants with "sepsis" had comparable levels of sepsis severity for evaluating the impact of antimicrobial treatment timings in subcategories of sepsis patients. Included are observational cohort studies, randomized controlled trials, and analyses of prospectively obtained data on the optimal timing of antibiotic administration. Not considered were meta-analyses, animal research, opinion pieces, small-scale studies, and editorial letters.

Participants Features

We documented the participant selection procedure, inclusion criteria, study duration and time period, study site (emergency room, intensive care unit, ward), study type, and sample size for each sepsis severity stratum. In addition, we collected data on the trial's baseline (i.e., the beginning of the investigation), the time intervals or cutoffs used to evaluate the efficacy of antibiotic treatment, and the primary outcomes of the study. In cases where microbiological documentation of infection was available, in vitro susceptibility of the causative pathogens was used as an analysis criterion, whereas in cases where clinical documentation of infection was available, antibiotic therapy management guidelines were used [12].

Subgroups Evaluation

Subgroup analyses were conducted to determine whether sepsis and septic shock had distinct outcomes. In addition, we conducted meta-analyses with stratified post-hoc analyses of trials to determine if antibiotics were administered appropriately.

Outcomes Assessment

All-cause mortality was the primary outcome measure. In all but five of the studies [13-17], confounding factors such as age, gender, Charlson index measurements, acute physiological and chronic health evaluation II (APACHE II), microbial entry site, sequential organ failure assessment (SOFA) score, hypotension, fluid restoration, lactate clearance, and vasopressor use were accounted for using multivariable analyses. Secondary outcomes included admission to the ICU, duration of the intensive care unit or hospital stay, progression of sepsis to septic shock, and death at predetermined intervals following hospital discharge.

Results

Studies Selection

**Figure 1:**
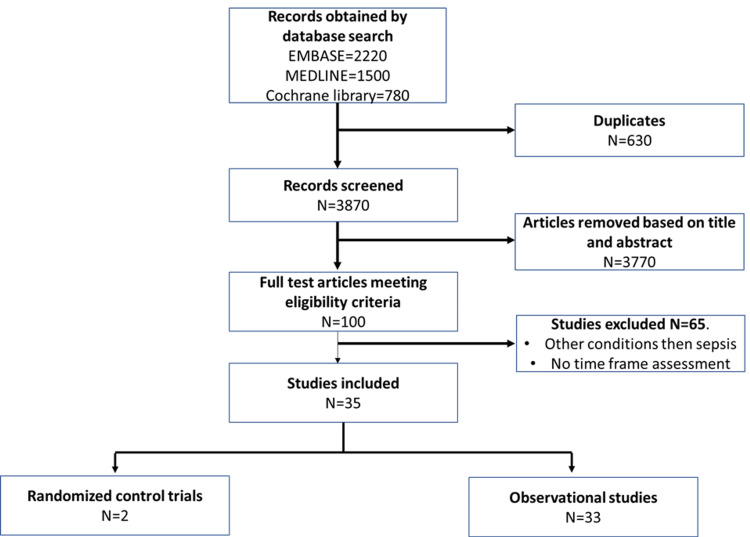
PRISMA flow diagram. PRISMA: preferred reporting items for systematic reviews and meta-analyses.

We decided to read the full versions of 100 articles out of a total of 4500 after reading the abstracts. On Cohen's kappa scale, there was a 0.81 agreement between the two reviewers [18]. After conducting a full-text search, we included 35 papers; one of these is an experimental trial study, while the remaining 34 are observation studies, including 14 prospective and 20 retrospective studies. Figure 1 depicts preferred reporting items for systematic reviews and meta-analyses (PRISMA) study selection strategy.

Studies Characteristics

Thirty-five included studies contain a total of 154,330 participants, with a median of 1058 participants per study, a minimum of 117 participants, and a maximum of 49,331 participants. From 1989 until 2020, there was a large window of opportunity for enrollment. Typically, research projects lasted 2.1 years (median) (min: 0.4, max: 15.5). Studies evaluating the timing of antibiotic medication included a variety of parameters, such as the starting period/time zero, the time cutoffs (with a minimum difference of two hours between each interval), and the time interludes. Twenty-one of the studies were conducted in emergency rooms, eight in ICUs, one in a ward, and five in a combination of these three locations. About 31% (51.094) of patients, 61% (39.6%) of patients, and 8% (13,444) of patients, respectively, suffered from septic shock or sepsis with or without organ failure. In nine studies, patients with septic shock, severe sepsis, and sepsis comprised 18.6% of the total population. Mortality was the endpoint in all but one study, with adjusted mortality used in 83% (n=29), all-cause crude mortality in 14.3% (n=5), and a combination of adjusted and unadjusted death in one study. About 68.6% of studies (n=24) evaluated mortality at the conclusion of the ICU stay, 20% of studies (n=7) at day 28 or day 30, 8.6% of studies (n=3) at various time intervals, and 2.8% of studies (n=1) after one year.

Cause-and-Effect Relationship Between Antibiotic Administration Time and Death

In 68.6% of studies (n=24) [4,6,14,16,19-31], the time at which antibiotics were administered was associated with in-hospital mortality, 14.2% (n=5) [29,32-35] reported death at other times, and 2.8% (n=1) [17] studies demonstrated mortality associated with hospital or ICU length of stay. In 40% (n=14) of the included studies, a wide variety of time cutoffs (Table 1) were associated with the participants' outcome, whereas in 23% (n=8) of the studies, an hourly time interval up to 24 hours was associated with the participants' outcome. Ten studies [14,15,21,25,27,29,36-39] used the moment of ED admission as their starting point; four of these studies [21,25,27-29] discovered a correlation between time cutoffs and patient outcome in multivariate analyses. In nine studies [4,13,20,22,31,32,35,40,41], the onset of symptoms or signs of sepsis was designated as the starting period, and in five of these studies [4,20,22,31-35], the multivariable analysis revealed a correlation between death and specific time cutoffs.

**Table 1:** Study characteristics. AB: antibiotics, APACHE II: acute physiological and chronic health evaluation II, RCT: randomized control trial.

Author, year	Study type	Sample size	Setting	Sepsis severity types	Initial time of AB administration	AB time intervals and cutoff	Death place and point	Mains discoveries
Alam et al., 2018 [13]	RCT	2631	ED	Septic shock, severe sepsis, and sepsis	Identification of shock by the paramedic	1-4 h and >4 h	Crude death rate at day 28 due to all causes	There was no correlation between the use of antibiotics in the prehospital setting and 28-day mortality.
Ballester et al., 2018 [14]	Retrospective observational	153	ED	Septic shock, severe sepsis, and sepsis	ED admission	Median	In hospital	Door-to-antibiotic time was correlated with death.
Castano et al., 2019 [37]	Prospective observational	705	ED	Septic shock and severe sepsis	ED admission	Hourly	In hospital	There was no correlation between antibiotic administration delays and hospital mortality.
De Groot et al., 2015 [36]	Prospective observational	1168	ED	Sepsis	ED admission	0-1, 1-3, and >3 h	After 28 days outside the hospital	Time to antibiotics is not associated with either out-of-hospital survival days or fatality.
Gaieski et al, 2010 [19]	Retrospective observational	261	ED	Septic shock and severe sepsis	ED triage	1-5 h	In hospital	Triage to proper antibiotic delivery time (within an hour) and in-hospital mortality.
Husabo et al, 2020 [34]	Retrospective observational	1559	ED	Sepsis	ED triage	1-4 h interval, >4 h	Crude death rate at day 30 due to all causes	Antibiotics are given within two to three hours after ED entry reduced mortality compared with those given two or three hours later.
Jalili et al., 2013 [15]	Prospective observational	145	ED	Sepsis	ED admission	1-2 h, >2 h	Sepsis associated death	Time from arrival at the hospital to the administration of antibiotics has been linked to death, but only among those with high APACHE II scores.
Joo et al., 2014 [25]	Retrospective observational	591	ED	Septic shock and severe sepsis	ED admission	0-3 h vs. >3 h	In hospital	Timely antibiotic treatment (within three hours) has been linked to better outcomes (including patient mortality, recovery from organ failure, and reduced hospital length of stay).
Kim et al., 2018 [26]	Retrospective observational	117	ED	Septic shock and severe sepsis	ED triage	0-3 h vs. >3 h	In hospital	Correlation between antibiotic administration time and hospital mortality after triage.
Ko et al., 2020 [42]	Prospective observational	2229	ED	Septic shock	ED triage	0-3 h vs. >3 h	In hospital	Propensity score analysis shows an inverse relationship between antibiotic administration time (1 h) and hospital mortality. Hospital mortality was not linearly related to waiting times.
Liu et al., 2017 [21]	Retrospective observational	35,000	ED	Septic shock, severe sepsis, and sepsis	ED admission	0-6 h, 30 min interval	In hospital	Antibiotic treatment delays of up to six hours were linked with higher adjusted odds ratios for hospital mortality across all sepsis severity strata.
Londoño et al., 2018 [27]	Prospective observational	884	ED	Septic shock and severe sepsis	ED admission	1-24 h	In hospital	Antibiotics given within one or three hours have been linked to lower death rates.
Peltan et al., 2019 [29]	Retrospective observational	10,811	ED	Septic shock and severe sepsis	ED admission	1-6 h, Hourly interval	In hospital and up to one-year death	Time from entering the hospital to receiving antibiotics (three hours) is related to an increased adjusted risk of dying within a year.
Ryoo et al., 2015 [32]	Retrospective observational	426	ED	Septic shock	Shock diagnosis	1-5 h, Hourly interval	28th Death	There was no correlation between antibiotic administration delays of one hour or more.
Puskarich et al., 2011 [7]	Control trial	291	ED	Septic shock	Shock diagnosis	1-6 h, Hourly interval	In hospital	There was no correlation between the time it took to start antibiotic treatment (up to six hours) and the patient's risk of dying while hospitalized.
Seok et al., 2020 [39]	Prospective observational	482	ED	Septic shock and sepsis	ED admission	0-3 h, Hourly interval	Death at the 7, 14, and 28th day	Overall and subgroup studies, including (patients with septic shock or with suitable antibiotics), found no correlation between delay to antibiotics and outcomes.
Seymour et al., 2017 [5]	Retrospective observational	49,331	ED	Septic shock and severe sepsis	Contact with paramedics	0-12 h, Hourly	In hospital	Increased risk-adjusted in-hospital mortality was seen in patients whose antibiotic treatment took longer to begin.
Seymour et al., 2017 [30]	Retrospective observational	2683	ED	Septic shock and sepsis	Contact with paramedics	0-12 h, Hourly	In hospital	Hospital mortality is increased when antibiotic treatment is delayed in the emergency department.
Anser et al., 2021 [16]	Retrospective observational	261	ED	Septic shock, severe sepsis, and sepsis	ED admission	0-3 h vs. >3 h	In hospital	Patients who got antibiotics within three hours had a lower risk of dying while hospitalized.
Whiles et al., 2017 [23]	Retrospective observational	3929	ED	Severe sepsis	ED admission	1-24 h, Hourly	In hospital	Mortality and the development of septic shock were linked to a delay in the delivery of antimicrobials.
Wisdom et al., 2015 [43]	Retrospective observational	220	ED	Severe sepsis and sepsis	ED triage	1-6 h, Hourly interval	In hospital	No connection between time from triage to delivery of antibiotics and death in the total population; the trend among patients with severe sepsis who got antibiotics after six hours.
Abe et al., 2019 [40]	Prospective observational	1124	ICU	Septic shock and severe sepsis	Shock diagnosis	0-1440, Continuous intervals	In hospital	No correlation was found between the time of antibiotic delivery (1 h, 3 h, or time as a continuous variable) and hospital mortality.
Bloos et al., 2014 [44]	Prospective observational	1011	ICU	Septic shock and severe sepsis	ED admission	1-6 h, Hourly interval	Crude death rate at day 28 due to all causes	There was no linear correlation between the time it took to start antibiotics and the 28-day death rate.
Bloos et al., 2017 [33]	Prospective observational	4183	ICU	Septic shock and severe sepsis	Initial organ failure	Hourly	Crude death rate at day 28 due to all causes	The risk of dying within 28 days rises by 2% for every hour that antibiotic treatment is delayed.
Ferrer et al., 2009 [24]	Prospective observational	2796	ICU	Septic shock and severe sepsis	Symptom identification	1-6 h, Hourly interval	In hospital	Hospital mortality was reduced when antibiotics were started early (within one hour compared to no treatment within six hours).
Kumar et al., 2006 [6]	Retrospective observational	2154	ICU	Septic shock	Initiation of hypotension	1-36 h	Outside	Relationship between antibiotic administration delay and death assessed by the hour.
Peng et al., 2018 [22]	Retrospective observational	541	ICU	Septic shock and sepsis	Shock diagnosis	1-48 h	In hospital	Increased mortality in the intensive care unit and across the hospital is linked to antibiotic treatment delays.
Suberviola Canas et al., 2015 [31]	Prospective observational	342	ICU	Septic shock	Documentation of shock	1-6 h, Hourly interval	In hospital	Delay in antibiotic therapy is associated with higher mortality.
Yokota et al., 2014 [41]	Retrospective observational	1279	ICU	Septic shock and severe sepsis	Severe sepsis diagnosis	0-1 h	In hospital	Death rates were not lower after antibiotic treatment.
Ascuntar et al., 2020 [38]	Prospective observational	2454	ICU + ED	Septic shock and sepsis	ED admission	1 and 3 h	In hospital	There was no correlation between antibiotic administration time (within one hour or three hours) and hospital mortality.
Ferrer et al., 2014 [20]	Retrospective and prospective observational	17,990	ICU + ED	Septic shock and severe sepsis	ED triage	1-6 h, Hourly interval	In hospital	Hospital mortality is higher when antibiotics are not given within two hours.
Zhang et al., 2015 [17]	Retrospective observational	1058	Wards	Septic shock and severe sepsis	In vitro AB susceptibility	0-24 h, Hourly	In hospital	Longevity of stay in the intensive care unit or hospital as a function of time to adequate antibiotic medication (one-hour increments).
Pruinelli et al., 2018 [4]	Retrospective observational	5072	Wards	Septic shock and severe sepsis	Sepsis diagnosis	0-6 h, Hourly interval	In hospital	There is a link between antibiotic delivery times longer than 125 minutes and death.
Nygard et al., 2014 [28]	Prospective observational	220	Wards	Severe sepsis	ED admission	1-6 h, Hourly interval	In hospital	Antibiotic treatment delays of six hours or longer have been linked to an increase in mortality.
Lueangarum and Leelarasamee, 2012 [35]	Retrospective observational	229	Wards	Septic shock, severe sepsis, and sepsis	Sepsis diagnosis	1-6 h, Hourly interval	Crude death rate at day 28 due to all causes	Greater than a three-hour lag in antibiotic delivery is related with increased mortality.

Antibiotic Treatment Appropriateness

In 54% (n=19) of the included studies, antibiotic treatment was deemed appropriate for 13% (n=20,062) of the total reviewed participants. About 83% (16,652) of these 20,062 participants received the appropriate antibiotics. Eleven of these 19 studies utilized in vitro susceptibility criteria, while the remaining eight utilized a combination of clinical and microbiological factors. In ten of these nineteen trials, researchers discovered a correlation between the antibiotic delay and mortality rates for delays of one hour, three hours, six hours, and longer. In three studies [6,17-42], mortality in patients with septic shock was linked to delays of one hour or more. In all but one of the trials, multivariate analyses were conducted [6].

Sepsis and Septic Shock Investigations

Following this, we analyzed studies involving patients with septic shock or sepsis to determine how the timing of antibiotic administration impacted patient outcomes.

Sepsis

About 57.1% (n=20) of studies included participants with sepsis or organ failure without sepsis; six out of twenty studies involving 24,281 participants evaluated the association between antibiotic administration timing and mortality [13,21,23,28,39-43]. In three studies involving 92.0% of patients, a delay of >6 hours in administering empirical antibiotic medication was associated with an increase in mortality rate [21,23-28]. A delay in antibiotic treatment increased the incidence of septic shock by 8%, according to one study [23]. In the remaining three studies, early antibiotic treatment did not reduce mortality.

Septic Shock

About 60% of studies (n=21) included patients with septic shock; eleven of these studies examined the effect of antibiotic administration timing on mortality in 12,756 patients [6,7,17,21,31,32,38,40-42]. In five trials involving 79.6% of patients, starting antimicrobials >3 hours after triage in the ED was associated with an increase in in-hospital mortality or ICU length of stay [6,17,21,31-42]. In the remaining six studies, no correlation was found between early antibiotic treatment and prognosis.

Discussion

Sixty-six percent of the studies included in this meta-analysis discovered a correlation between prompt antibiotic use and decreased mortality (Table 1). Nonetheless, time metrics for antibiotic administration were significantly associated with patient outcomes, despite thresholds ranging from one to six hours, depending on the study. Studies [5,6,21,29-33] that utilized risk-associated linear models spanning 6 to 12 hours and found an increase in death rate for each hour of delayed antimicrobial administration were likely influenced by increased mortality odds. Importantly, the largest studies [36,40-44] did not find any correlation between the timely administration of antibiotics and improved patient outcomes. On the basis of the available evidence, it is therefore impossible to provide highly informed recommendations regarding the timing of antimicrobial administration in sepsis patients.

This review confirms the findings of two other meta-analyses and systematic reviews, but with three times the number of studies [30,45,46]. The use of antibiotics within an hour of identifying shock or sepsis or within three hours of triage in the ER did not improve survival, according to an analysis of 11 trials conducted by Sterling et al. Similarly, a meta-analysis of 13 trials found no difference in mortality among participants who received antimicrobials within one to three hours of the onset of sepsis [46]. In contrast, an analysis of 10 studies conducted by Johnston et al. revealed that participants who received antimicrobials within one hour had a 33% lower risk of mortality. However, after accounting for a number of covariables, one study reported a 7.5% increase in the death rate, significantly distorting the overall results. It is also problematic that patients who received antibiotics at widely variable intervals (>1-6 h) after being admitted to the emergency department were grouped together and analyzed as a whole in a number of studies [47].

Protocols for the timing of antibiotic administration should be stratified according to the severity of sepsis, which is widely recognized as an important factor in patient outcomes [48]. Antibiotics should be administered immediately to patients in septic shock due to the high mortality rate associated with the condition. Due to the fact that sepsis occurs on a continuum with no discernible zone of infrequency, it is difficult to provide treatment with high prognostic validity across a wide range of disease probabilities [49]. Although postponements were also associated with a higher mortality rate among patients with sepsis, no significant threshold period was derived from the available data for this category. In theory, antibiotic treatment delays for patients with suspected sepsis may be modified according to the severity of their illness. A recent analysis of the prognosis of patients with bacterial infections of varying causes and severities supports this approach [50].

Patients in septic shock or with bacterial meningitis should get antibiotics immediately, but the authors found no indication that outcomes worsened when medication was delayed for four to eight hours in participants with moderate illnesses. The dangerous development of sepsis into septic shock is a dangerous cost of delaying treatment. Only one of the papers we looked at even touched on this, and its prevalence was determined to be 8%. The mortality rate would rise from 10% in sepsis to 12.4% in septic shock, taking into account the fact that sepsis has a 10% mortality risk and a 40% probability of death in septic shock. It's a fine balancing act to determine whether the possible benefit of the morbidity criterion is worth the higher risk of death. Participants with suspected sepsis who are not in crisis and have a low risk of infection may be an ideal patient group in which to test the feasibility and safety of close clinical surveillance followed by rapid antibiotic start upon evidence of infection.

The strengths of the present review include its large sample size, careful evaluation of eligibility that increases the consistency of the findings, subgroup analysis conferring to sepsis severity determined post-hoc, and priori post-hoc evaluation of studies emphasizing the appropriateness of antimicrobial treatment. The majority of the research's limitations stem from the observational methodology's inherent bias. The absence of data on the optimal timing of antibiotic treatment and the use of varying descriptions of time zero may also impact the reliability of the results, as may the presence of individuals with varying sepsis durations and severity levels. Cumulative odds ratios or relative risks are useful, but their predictive power is severely limited due to the lack of data on the sample size of participants in each sepsis severity category provided by the majority of studies. The absence of any discussion regarding the implementation of therapeutic drug monitoring and source control is glaring in these studies. These essential characteristics should be investigated in subsequent research.

## Conclusions

In two-thirds of the clinical trials that were analyzed for this meta-analysis, there was a connection between early antibiotic treatment and mortality. However, there was a significant amount of heterogeneity in the time metrics that were associated with early antibiotic allocation and death. Furthermore, there was not a clear time threshold that emerged, either in the entire research population or in the subgroups of trials that contained participants with septic or shock sepsis.
